# Transport of Live Cells Under Sterile Conditions Using a Chemotactic Droplet

**DOI:** 10.1038/s41598-018-26703-y

**Published:** 2018-05-30

**Authors:** Silvia Holler, Carlotta Porcelli, Ioannis A. Ieropoulos, Martin M. Hanczyc

**Affiliations:** 1 Laboratory for Artificial Biology, Centre for Integrative Biology (CIBIO), University of Trento, 38123 Trento, Italy; 2 Chemical and Biological Engineering, University of New Mexico, MSC01 1120, Albuquerque, NM 87131-0001 USA; 3 Bristol BioEnergy Centre, Bristol Robotic Laboratory, Block T, UWE, Bristol, Coldharbour Lane, Bristol, BS16 1QY UK

## Abstract

1-Decanol droplets, formed in an aqueous medium containing decanoate at high pH, become chemotactic when a chemical gradient is placed in the external aqueous environment. We investigated if such droplets can be used as transporters for living cells. We developed a partially hydrophobic alginate capsule as a protective unit that can be precisely placed in a droplet and transported along chemical gradients. Once the droplets with cargo reached a defined final destination, the association of the alginate capsule and decanol droplet was disrupted and cargo deposited. Both *Escherichia coli* and *Bacillus subtilis* cells survived and proliferated after transport even though transport occurred under harsh and sterile conditions.

## Introduction

The development of soft matter systems that mimic the behavior of living systems^1^ can be useful in studying related processes in natural living systems and also in pioneering new materials^2^ as well as technological designs^3^. Soft matter systems can be driven out of equilibrium and can show large responses to external stimuli^4^. The switch from equilibrium to non equilibrium systems can be driven through, for example static external fields, externally imposed physical shear flows, or chemical potential. By increasing the distance from the equilibrium ground state, the complexity of the system increases and active states emerge. For example, self motion is an emergent behavior, as shown for artificial micro swimmers^5^. Some self-moving systems also show the ability to move directionally in response to chemical signals in the environment and therefore such systems are capable of chemotaxis^6^.

We have been developing several types of self-moving and transforming droplet systems^6^. We have so far focused on taxis^6^, shape change^7^, maze-solving^8^ (see also^9^), and rudimentary fission-fusion cycles^10^. Through such studies, several key processes of living systems can be recapitulated in highly simplistic chemical and physical systems, albeit abstract and artificial in reference to the natural living systems^11,12^.

Given the inherently artificial corporeality of these systems, the development of an interface between, for example, a tactic droplet and a living cell becomes a challenge. The experimental conditions that support the necessary fluid dynamics and chemical reactivity for self-motion in droplets may be detrimental for the sustenance and proliferation of living organisms. The harsh conditions could include chemicals such as nitrobenzene, surfactants at levels to solubilize cell membranes, and high pH solutions (up to units of 12). Such conditions may severely affect the viability of living cells. Although there are many droplet systems that have been reported as transport systems, none have shown compatibility with hosting and transporting living cells outside of aqueous droplets in a microfluidic platform^9,13^.

In order to integrate living cells into the chemical system, a protective shell or matrix can be used. Sodium alginate is a compound with a broad use in biomedical applications and bio-engineering^14^. Typically alginate is prepared in water and cross-linked forming a hydrogel. Alginate hydrogels are exploited for different applications: wound healing, drug delivery, *in vitro* cell culture and tissue engineering. Alginate produces safe and reliable effects in many applications, for example in the treatment of type 1 diabetes^15^ and treatment of urinary incontinence and vesicoureteral reflux^16^. In addition, alginate hydrogels can be applied to diverse applications when modified^17^.

However, alginate is by composition too hydrophilic for stable integration into the hydrophobic 1-decanol droplets. Several alternatives for using chemically modified hydrophobic alginate exist and require chemical synthesis and purification or extreme chemical modification. For example cold plasma treatment could be used to create alginate surfaces with hydrophobic properties^18,19^. In addition, several alginate derivatives have been synthesized to create an amphiphilic alginate that requires the chemical modification of the alginate backbone by alkyl chains and other hydrophobic moieties^14^.

Alternatively we could consider a different type of protective capsule, for example based on hydrophobic coatings used for liquid marbles^20^ or more sophisticated multilayer capsules that could exploit the properties of self-assembled short peptides^21^. Such alternative capsules could be advantageous and allow for more consistent survival of many different types of cells in our transport system.

In this paper we experimented with a simple solution to physically integrate an alginate capsule containing live cells into hydrophobic droplets by adding a surfactant during the hydrogel crosslinking step. This method resulted in an alginate hydrogel with definable hydrophobicity by simply titrating the amount of surfactant added. This solution has the added benefit of being easily dissolved allowing for release of the cargo.

Here we describe a self-moving chemical droplet system for the controlled transport and deposition of living cells. We based the experiments on the chemotactic motion of 1-decanol droplets in decanoate solution at high pH^8^. We tested various cells for compatibility with the chemical system including *Escherichia coli (E. coli)*, *Bacillus subtilis (B. subtilis)*, *Vibrio fischeri (V. fischeri)* and *Saccharomyces cerevisiae (S. cerevisiae)*. The cells were temporarily placed in a biocompatible capsule as a protective environment but also as a measurable and well defined unit of cargo for transport. We then used the self-moving 1-decanol droplets as vehicles to transport the hydrophobic alginate capsules. A droplet with one or more alginate capsules was able to move chemotactically in salt gradients. After transport, the association between the alginate capsule and the decanol droplet was disrupted, and the alginate capsules were harvested from the system. We show that *E. coli* and *B. subtilis* cells were alive and proliferated following the droplet-mediated tactic transport, under otherwise sterile conditions.

## Results

### Chemotactic droplet experiment

**Figure 1:**
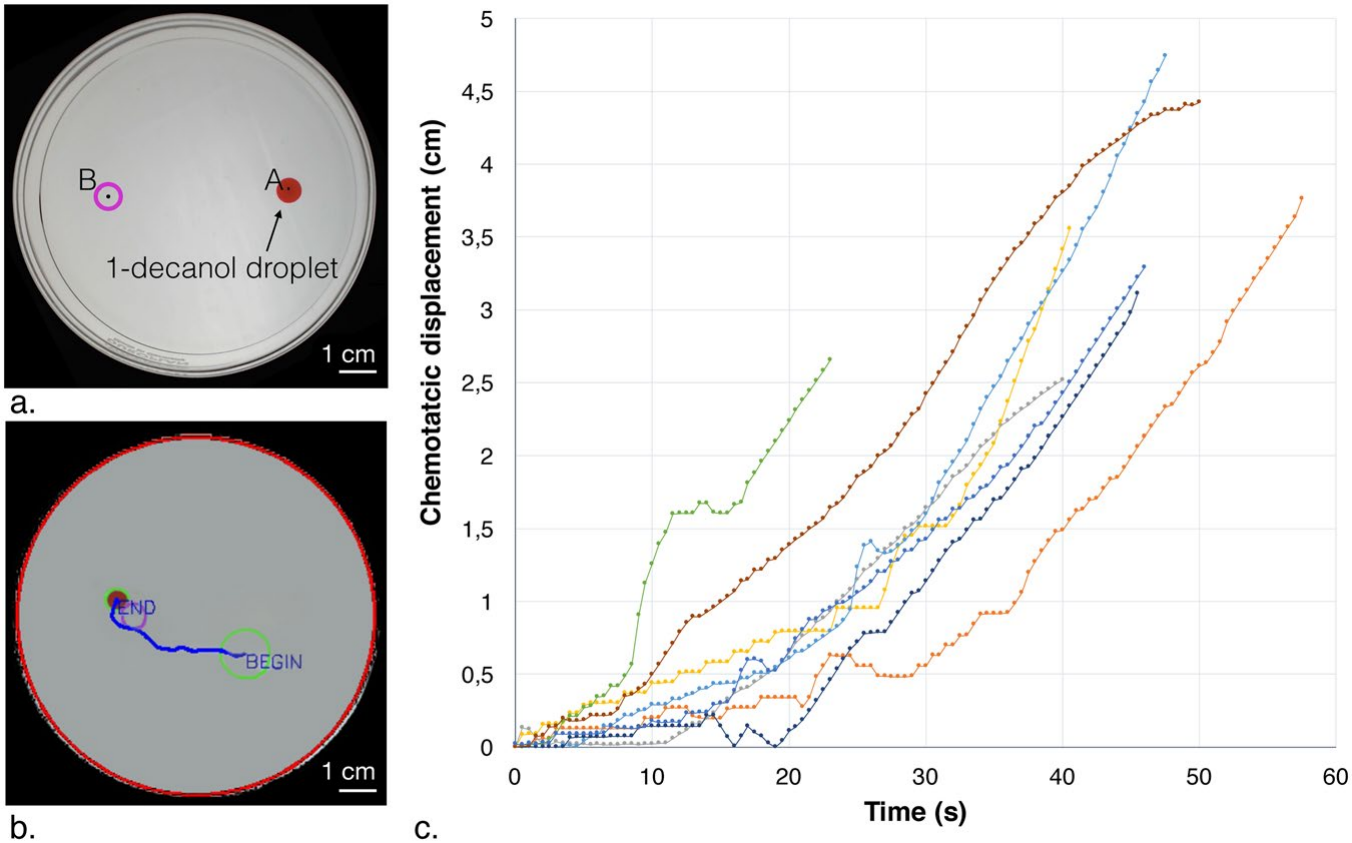
General design of droplet-mediated transport experiment and tracking. (**a**) The diameter of the glass Petri dish is 90 mm. 9 ml of 5 mM decanoate pH 11 solution was added to the dish. The 1-decanol droplet, colored with Oil Red O for easy visualization, was added at position A. Salt is added at position B to create the gradient. (**b**) The migration of the droplet is monitored with a video camera and the lateral movement is recorded, see Methods. The droplet path is shown as a blue line generated by the tracking algorithm. (**c**) Videos of eight droplet experiments were analyzed for the time resolved displacement (y-axis) over time. Each point represents a 0.5 second interval. Each droplet is represented by a different colored line.

Chemotaxis, the directional movement of cells or organisms in response to chemical gradients, can be reproduced using chemical systems^9,22,23^. The taxis system we used composed a 1-decanol droplet and a surrounding environment of decanoate solution (typically 5 mM, pH 11-12). A chemical gradient is then created with the addition of sodium chloride (3 M NaCl) and the droplet moves chemotactically towards the source of the gradient. We tracked the droplet movement for eight experiments under standard conditions (decanoate 5 mM at pH 11; see Fig. 1) and the mean velocity of droplets after the addition of 450 *μ*l of 3 M NaCl is 0,049 ± 0,007 cm/sec.

The explanation of this motile droplet system has been published in^8^ describing how changes in interfacial tension govern the motion and direction on the droplets. The effect of increasing NaCl on the interfacial tension of the droplet as employed in this paper is shown in Supplementary Fig. S1. The test system is depicted in Fig. 1: A droplet of 1-decanol is placed at the starting point A, and then the salt is deposited in target position B. This shows that the directional motion of the droplet is dependent on the salt gradient, as previously described^8^ and is consistent with the system finding the lowest energy state (Supplementary Fig. S1). In addition, this kind of system can be exploited to transport light objects such as a conductive copper wire^24^. Next we tested if this droplet system can be used to transport live cells protected in alginate capsules.

### Alginate capsule design

**Figure 2:**
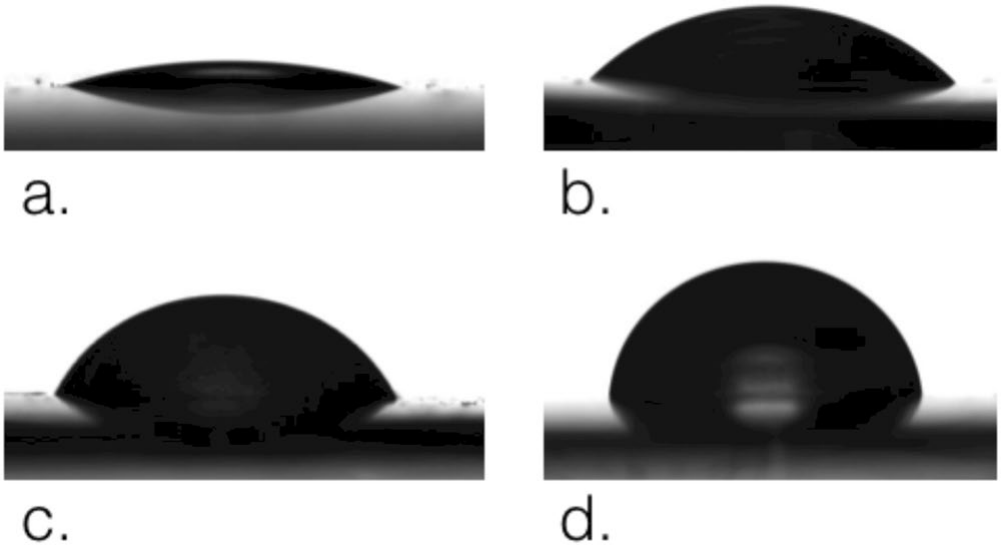
Images of contact angle of water on alginate. Alginate was dissolved in different aqueous solutions: water pH 7 (**a**), water pH 12 (**b**), decanoate 10 mM (**c**) and decanoate 25 mM (**d**). The contact angles for the sessile drop of water (pH 7) were as follows: a. water pH 7: 14,5° ± 1,92°; (**b**). water pH 12: 38,62° ± 2,06°; (**c**). decanoate 10 mM pH 12: 61,02° ± 2,96°; (**d**). decanoate 25 mM pH 12: 86,04° ± 2,01°. Contact angle value for alginate dissolved in 5 mM decanoate pH 12 was similar to alginate dissolved in water pH 12 (39,75° ± 4,6° Supplementary Fig. S3).

We prepared the alginate capsules using standard protocols starting from a solution of sodium alginate using calcium chloride (CaCl2) to crosslink the matrix. This produced completely crosslinked alginate capsules of roughly 2 mm diameter (see Supplementary Fig. S2). Initial trials showed that the inherent hydrophilicity of the alginate hydrogel was incompatible with the 1-decanol droplet. The capsule created from alginate prepared in water would not stably associate with the droplet and would be shed into the water phase instantly. We then modified the hydrophilicity of the alginate by preparing the hydrogel in water supplemented with varying concentrations of decanoate surfactant. Capsules prepared with alginate supplemented with 5, 10 or 25 mM decanoate pH 12 showed an increasing association affinity with the decanol droplet. We measured the contact angle of a water droplet (4 *μ*l) on alginate hydrogel formed with increasing concentrations of decanoate surfactant (also at pH 12). After crosslinking the hydrogel surface was washed with *Milli-Q* water and the surface dried briefly using nitrogen gas. We demonstrated the increase in hydrophobicity of the alginate surface with increasing decanoate concentration, as shown in Fig. 2. The increased hydrophobicity of the alginate allowed for such an alginate capsule (about 1 mm in diameter after drying) to be physically embedded in a 20 *μ*l volume 1-decanol droplet in decanoate solution. Contact angle values can be found in Supplementary Fig. S3. We note that this is a transient surface modification. This is evidenced by long term contact angle experiments that show that pure water droplets on alginate supplemented with decanoate surfactant eventually wet the surface, indicating that the surfactants ultimately leave the hydrogel interface and distribute throughout the system.

### Alginate capsule transport with live cells

**Figure 3:**
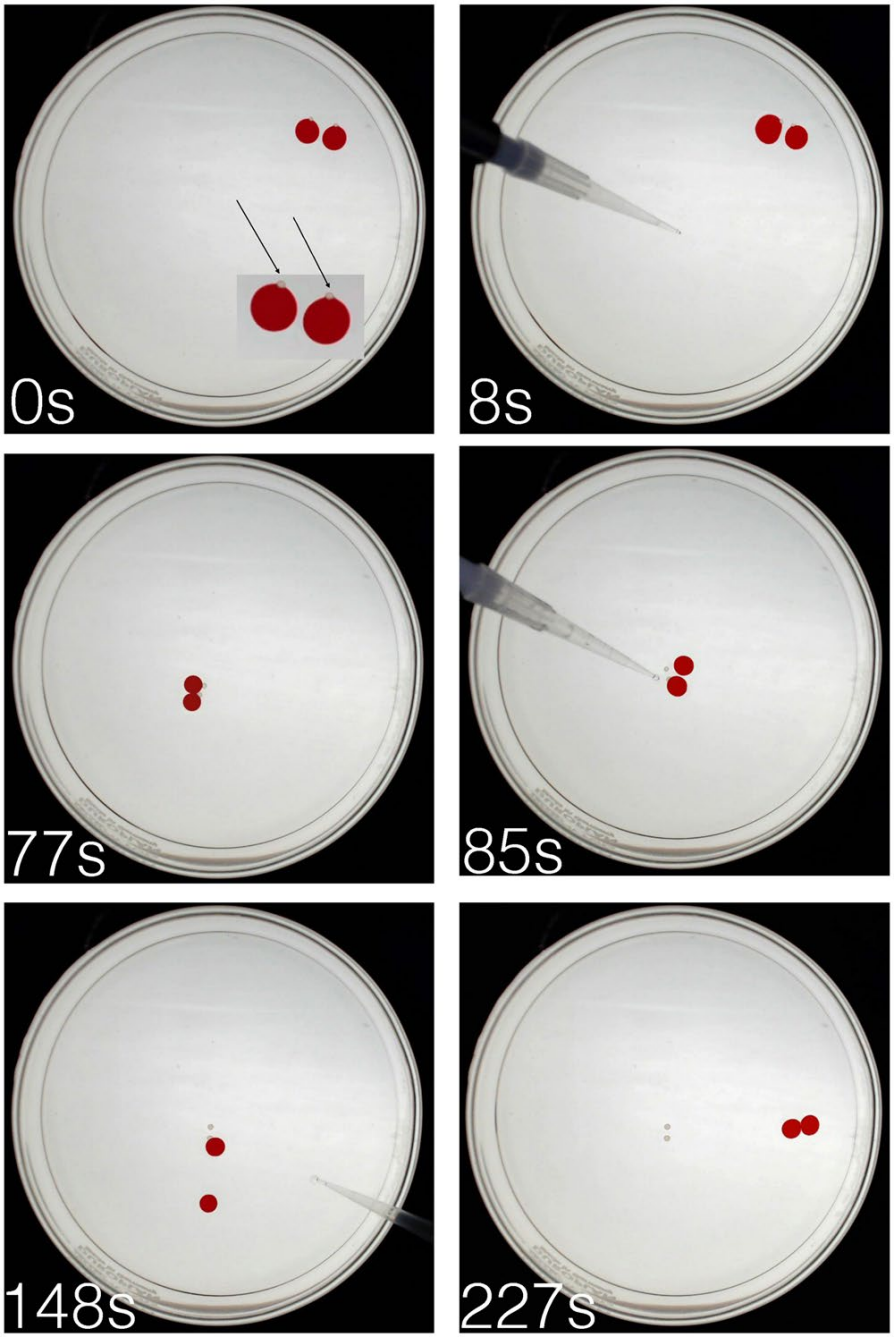
Decanol droplet transport of alginate capsule cargo. The diameter of the glass Petri dish is 90 mm. Two 1-decanol droplets are placed in the Petri dish, each containing an alginate capsule of 1 mm diameter (the small capsules are indicated in the inset with arrows). 0 seconds (sec, s): droplets with capsules placed in Petri dish; 8 sec: salt gradient added with pipette (visible); 77 sec: end of droplet migration towards salt source; 85 sec: 0.2 M decanoate addition by pipette (visible) and capsule deposition; 148 sec: second salt addition; 227 sec: droplet migration towards second salt source. See also Supplementary movie 1.

Figure 3 shows how droplets of 1-decanol can be used to transport and deposit alginate capsules in our system. A capsule of alginate is placed manually inside each droplet of 1-decanol. The droplets successfully transport both capsules along the salt gradient. When the droplet reaches the target destination, the alginate capsule cargo is released through the manual addition of a decanoate solution with high molarity (0.2 M). This solution changes the interfacial tension of the droplet and the physical interaction between the droplet and the alginate cargo is disrupted. This allows the cargo to be dropped in a specified position as shown. For a video of this system, see Supplementary Movie 1 25x https://www.youtube.com/watch?v=zCB2bPhFoCI.

We see a relationship between the droplet volume and its carrying capacity. For example, a droplet of 10 *μ*l can transport only 1 capsule efficiently. Roughly with an increase of 10 *μ*l in droplet volume, an additional capsule can be stably transported.

### Viability of cells after the encapsulation and transport

**Figure 4:**
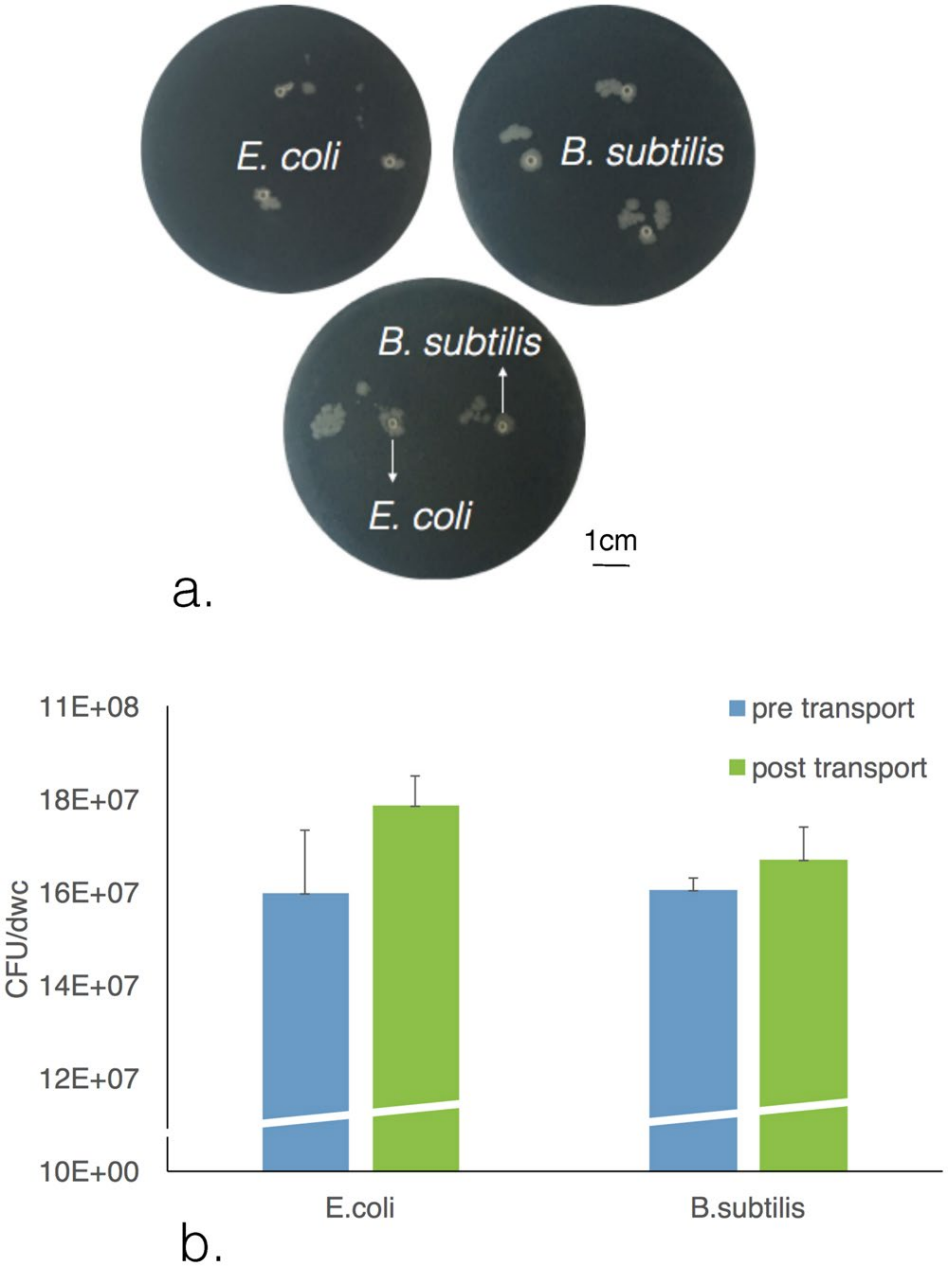
Bacterial encapsulation, transport and survival. Panel a shows cell growth from transported capsules placed on grown media in Petri dishes. Panel b shows a bar plot that represents the Colony Forming Units (CFU) of each of 10 capsules pre and post transport. CFU values are normalized for the dry weight of the capsules (dwc) (error bars correspond to standard errors for experimental triplicates).

We tested four different kinds of organisms for encapsulation and transport: three bacteria *E. coli*, *B. subtilis* and *V. fischeri* and one eukaryote, *S. cerevisiae*. To test the sterility of the system, we performed a direct incubation of each organism in 1-decanol oil and in the decanoate aqueous solution, see Methods. Although a few *E. coli* and *B. subtilis* survive for 5 minutes in the 5 mM decanoate solution, none of the organisms survived when placed directly in 1-decanol, confirming the sterility of the system. We tested for the viability of each of the four organisms after being encapsulated inside the alginate capsules and then after capsule transport with the droplet. For encapsulation, the cells were added to the cross-linking step of alginate capsule formation along with their appropriate growth media and the decanoate for modifying the hydrophobic property of the alginate (see Methods). For each organism three replicates of ten such capsules were created, weighed, dissolved and plated on growth media to check for the survival of the organisms during the encapsulation step. In addition, individual capsules were added to 1-decanol droplets, transported, released and then harvested for assessment. Three replicates of ten transported capsules were dissolved and serial dilutions were performed for survival assessment and quantification of the cells. Both *E. coli* and *B. subtilis* survived the encapsulation steps as well as the transport and harvesting step under these conditions (Fig. 4). Additionally, for visualization purposes, we show that single alginate capsules post transport containing *E. coli* or *B. subtilis*, manually placed on Luria-Bertani (LB) agar plates (Fig. 4a), showed growth and colony formation.

In contrast we found that both *V. fischeri* and *S. cerevisiae* were more sensitive to such manipulations and did not consistently survive to the end of the transport step. However, we detected that *V. fischeri* survived at least the encapsulation step as evidenced by the autoluminescence (Supplementary Fig. S4). For yeast, the cells survive both the encapsulation and droplet-mediated transport. However the survival of the yeast is not consistent even within the same set of replicate experiments and therefore the results are not quantifiable. Summary of all cell viability assays is found in Table 1.

**Table 1:** Summary of cell viability tests.

	Direct addition to decanol	Direct addition to decanoate	Encapsulation	Transport
V. fischeri	no growth	no growth	growth	no growth
E. coli	no growth	growth	quantified growth	quantified growth
B. subtilis	no growth	growth	quantified growth	quantified growth
S. cerevisiae	no growth	no growth	growth	growth
Capsule only (control)	no growth	no growth	no growth	no growth

### Confirmation of sterile and aseptic conditions

We confirm that at least two cell types consistently survive both the encapsulation in alginate hydrogel and droplet-mediated transport. To confirm that this system of transport is aseptic and would prevent cell cross-contamination from capsule to capsule or from droplet to droplet within the same environment, we performed ten transport experiments for each organism (*B. subtilis*, *V. fischeri*, *E. coli* and *S. cerevisiae*). The test involved transporting two capsules per time using two droplets with only one capsule containing cells. The capsules not hosting cells however did contain the appropriate growth media. The capsules with and without cells were transported as described in the same experiment. After transport, each alginate capsule was harvested and placed in a falcon tube containing 5 ml of fresh media. The alginate hydrogel capsules spontaneously dissolve under these conditions. Following overnight incubation, only the tubes in which capsules containing cells were placed showed any cell growth.

In addition, each solution was spread on specific selective growth plates and only samples derived from capsules originally containing cells showed colony formation. We note that *E. coli*, *B. subtilis* and even *S. cerevisiae* consistently survived the experiment. All controls from capsules containing growth media but no cells showed no growth after transport. Therefore cross-capsule and cross-droplet contamination was prevented under these aseptic conditions. Results of all sterility, cross-contamination, and viability tests are summarized in Table 1 with control capsules labeled as ‘Capsule only’.

## Discussion and Conclusions

We have shown an integration of an artificial chemotactic droplet system with the directional transport of living cells. Since the chemotactic droplet system itself is functional under conditions that are incompatible with the viability of cells, a hosting capsule was developed that provided a favorable environment for the cells. In addition, the alginate hydrogel capsule supplemented with decanoate surfactant was able to interface with the droplet system, did not disrupt the droplet dynamics, was implantable and retrievable, and was able to release the live cell cargo for analysis and proliferation.

The droplet containing a capsule with live cargo could be manipulated with salt gradients several times with the capsule remaining stably attached to the droplet. In addition, several capsules can be stably fixed to a single decanol droplet. However, capsules placed in the droplet without drying or capsules larger than 1.5 mm in diameter tend to be dropped by the droplet during transport. This is presumably due to high water content of such capsules. Therefore the capsule size - more than the capsule number - is a limitation of the system.

Once the living cells are introduced to the system, they are subject to several steps of manipulation which could result in cell death: crosslinking in alginate with decanoate surfactant, drying of the capsules over time, placement in the toxic 1-decanol droplet, transport of the droplet with capsule over the course of minutes, release of the capsule from the droplet with additional surfactant, manual retrieval of the capsule from the system, and dissolution of the alginate capsule in fresh growth media. We therefore tested for cell viability at two key steps: after encapsulation and after transport, see Fig. 4 and Table 1. The best results were with *B. subtilis* and *E. coli* encapsulation and transport. These findings were reproducible and consistent. We see consistent viability of *V. fischeri* in the surfactant-alginate capsules due to detection of their quorum-sensing autoluminescence, see Supplementary Fig. S4. However, these cells do not consistently survive the recovery after encapsulation. Additionally we had mixed success with *S. cerevisiae* which survive encapsulation but do not consistently survive transport. Although we follow the protocol strictly and often perform many replicates (up to 10 per experimental condition, see Methods), there is some inherent noise in the system when transporting yeast that is not yet controlled. We therefore will investigate alternative encapsulation methods, such as those mentioned in the Introduction, to allow consistent viability of transported cells including yeast and other eukaryotic cells.

This type of system is presented for the first time and is a proof of principle demonstration including system dynamics and limitations. This represents an alternative platform to live cell manipulation using microfluidics^25^. In our case we take advantage of an open system that can be manipulated easily by hand but also using a robotic interface^26^. The structurally heavy architecture typically used in microfluidic systems for control is not strictly necessary when the actuation of the fluids is performed by the fluids themselves due to dynamic sensory-motor coupling^27^. Therefore such systems could be developed and applied to real world complex environments for the directed motion and delivery of cargo without the need for microfluidic superstructures and support systems. This proof-of-concept demonstration can be further developed for the transport of live cargo due to other controlling signals such as pH^6^, heat and light^28^. Transportation of cargo such as organisms or chemicals from one environment to another can also have wider applications. For example, a sulphur-reducing microbial community produces electroactive metabolites such as thiosulphate and hydrogen sulphide^29^ and in some cases, all the way to elemental sulphur^30^. These electroactive metabolites can be transported from the sulphur-contaminated environment to a non-contaminated environment and stored. Under the right conditions, this chemical energy can be converted to electrical energy^29^ and so in a way this transport system can result in a novel energy storage technology, customised for specific applications. We are currently developing this system towards these targeted outcomes. For example, we are developing a system for droplet mediated cargo transport under physiological conditions, which will expand greatly the potential applications of fluid droplet based transport.

## Materials and Methods

### Materials

All reagents for the chemotaxis experiments were supplied by Sigma Aldrich: decanoic acid, 1-decanol, Oil red O, sodium hydroxide, sodium chloride, sodium alginate, LB broth, agar, yeast extract, peptone and dextrose. Glass DURAN Petri dishes were supplied by Fisher, syringes from PIC and Glass slides from Prestige.

### Methods

Unless otherwise specified, experiments were carried out at room temperature.

#### Droplet motion analysis

For velocity analysis of droplet movement during chemotaxis, eight experiments were performed under standard conditions: 9 ml of decanoate 5 mM pH 11, 450 *μ*l of sodium chloride 3 M, 20 *μ*l of 1 decanol colored with 0.2 mg/ml Oil Red O. The tracking of the droplet position lasts for one minute and begins when the 1-decanol droplet starts to move from its initial position. The initial position is defined by a circle with a specific diameter of 3x the droplet diameter surrounding the point where the droplet is initially placed. This is necessary to avoid measuring initial fluctuations in droplet position that are common before the chemotactic movement begins. The tracking algorithm calculates the droplet velocity for each time frame and the mean of the calculated values was used to obtain the droplet mean velocity. The standard deviation was obtained from the 8 experimental replicates.

The eight experiments were tracked using EVOBOT (see https://real.itu.dk/people/afaina/attachment/20/) and the results were analyzed using a free software:’Tracker - Video Analysis and Model Tool’ (see https://physlets.org/tracker/). The video analysis has been performed by setting a reference measure from the’real object’, in this case the 90 mm of the Petri dish diameter. The changes of position of the droplet are registered in each frame. Data are shown in Fig. 1, where droplet displacement from the origin to the point of salt addition are plotted against time.

#### Surface tension measurement

1-decanol inverted pendant drop surface tension was analysed inside different water phases: decanoate 5 mM pH 11.5 and decanoate 5 mM pH 11.5 mixed 1:1 with NaCl 1, 3, or 5 M. Data were obtained for ten droplets (of 4-5 *μ*l) for each condition using a hooked needle (gauge 22), Theta-lite tensiometer Attension by Biolin Scientific (Nordtest Srl, Italy) and a high precision cell with optical path of 10 mm by Hellmark analytics.

#### Alginate hydrogel and capsule formation

Alginate hydrogel for contact angle analysis was first prepared with water or varying amounts of decanoate solution at pH 12. Sodium alginate was dissolved (5% w/v) in water (pH 7), water (pH 12, adjusted with 3 M NaOH), or decanoate solution (5, 10, 25 mM at pH 12, adjusted with 3 M NaOH). 1 ml of hydrogel with or without additional surfactant was placed on a glass slide (Prestige Micro slides 26 × 76 m), crosslinked with 1% w/v CaCl2 for 5 minutes and dried briefly with a stream of nitrogen gas. Pure water was then placed on the hydrogel and the contact angle measured for five droplets (of 4 *μ*l) for each condition using a Theta-lite tensiometer Attension, Biolin Scientific (Nordtest Srl, Italy). In parallel with the contact angle experiments, we prepared alginate capsules of roughly 2 mm diameter by crosslinking either alginate in water or alginate in the above surfactant solutions with 1% w/v CaCl2 (see Supplementary Fig. S2). Capsule size depends on the dimensions of the needle used for the pre crosslinking extrusion. We tested different types of needles and empirically determined that the 25 gauge, 16 mm needle produced the most consistent spheroidal alginate capsules of the desired diameter. These capsules were dried, using nitrogen gas flush for 5 minutes, dehydrated at room temperature for 3 hours, and then tested empirically for their association with a droplet of 1-decanol (20 *μ*l). After it was determined that alginate supplemented with 25 mM decanoate associated most stably with the decanol droplet and alginate capsules for transport were prepared. Sodium alginate (5% w/v) was dissolved in a decanoate solution (25 mM at pH 12, adjusted with 3 M NaOH). Cells to be transported were grown to a specific optical density (O.D.) and then pelleted and resuspended in fresh medium to a concentrated volume of roughly 1:10 (centrifugation: 1500 rpm 2 min for *S. cerevisiae*, 10000 rpm 10 min for *V. fisheri* and 15000 rpm 5 min for *B. subtilis* and *E. coli*). Nanodrop ND 2000 by Thermo Fisher was used to analyze optical density. The concentrated cells were then mixed with the alginate-decanoate solution (1:5 v/v). The alginate solution containing cells was loaded into a syringe (10 ml, needle 25 gauge, 16 mm) and dropped slowly by hand into 1% w/v CaCl2 for cross-linking. Round capsules were instantaneously formed. The capsules were cross-linked for five minutes and then washed once using *Milli-Q* water. Nitrogen gas was then flushed over the capsules for five minutes. Capsules were at this point consistent with typical alginate hydrogel crosslinked with CaCl2. We note that the outer surface of the capsule became more stiff after drying and capsules had an average diameter of 2 mm. Supplementary Fig. S2 shows four alginate capsules with 2 mm mean diameter on a black background. Optical image was taken using an iphone SE camera. The capsules were afterwards left to dry for three hours in an incubator at the temperature required for the growth of the organism encapsulated. The drying step was necessary to allow for surfactant assembly at the hydrogel surface to ensure consistent physical association between the alginate capsule and the decanol droplet. After this drying step, the average diameter of the capsules was 1 mm. After drying, the capsules were ready for transport. For capsules prepared without cells as controls, the same procedure was followed except instead of pelleted cells added to the alginate mixture, fresh sterile growth media was added. For capsules containing live cells, the system was prepared as follows: *Escherichia coli* (expressing pEGFP-N1) was taken from glycerol stock and grown overnight at 37 °C in LB broth. The same was done for *Bacillus subtilis* 168 strain. *Aliivibrio fischeri* MJ11 was resuspended in Luria-Bertani salt medium (LBS) and *Saccharomyces cerevisiae* BY4741 was instead resuspended in Yeast Extract-Peptone-Dextrose (YPD) and grown overnight at 30 °C. Before the encapsulation, the yeast strain BY4741 was diluted and reincubated for three hours to avoid the plateau of yeast growth. *V. fischeri* was instead resuspended in LB until O.D. 0.5. The grown cells were centrifuged and the pellets resuspended in fresh media to a concentrated volume of 1:10 for all four organisms. The obtained mixes were added to alginate 5%, mixed and capsules were prepared, as above.

#### Droplet-mediated alginate capsule transport

Glass Petri dishes of 9 cm diameter were filled with a volume of 9 ml 5 mM decanoate solution pH 11 (adjusted with 3 M sodium hydroxide (NaOH)). Droplets of (20 *μ*l) 1-Decanol were manually added to the system. Then dehydrated alginate capsules were placed manually on the decanol droplets. The chemotactic movement of the droplets was obtained through 3 M sodium chloride (NaCl) addition of 450 *μ*l, as previously described in Cejkova *et al*. 2016^8^. Alginate capsule deposition was effected though the addition of a decanoate solution (300 *μ*l) with a higher molarity (0.2 M pH 12) near the droplet. Droplets were then moved a second time through the addition of 600 *μ*l NaCl. Capsules were retrieved from the system using tweezers and further analyzed, as detailed below.

#### Cell viability

For assessing the viability of cells after encapsulation but before the transport, 3 biological replicates of 10 alginate capsules were created for each cell culture, dried and dissolved using sodium isocitrate 50 mM solution under continuous shaking for 15 minutes. The recovered samples were then centrifuged (1500 rpm 2 min for *S. cerevisiae*, 10000 rpm 10 min for *V. fisheri* and 15000 rpm 5 min for *B. subtilis* and *E.coli*). The pellets were resuspended in 1 ml growth media, plated after serial dilution (100 *μ*l, from 1:10 to 1:10^8^, three plating replicates each dilution) and incubated overnight. The plates were counted for colony forming units (CFU). Transport experiment were afterwards performed again with 3 biological replicates of 10 capsules prepared from the growing cell cultures and transported for an average time of 5 minutes. After transport, the capsules were washed with fresh media, dissolved (using sodium isocitrate 50 mM solution) and centrifuged (as above). Dilutions in appropriate growth media (1 ml) were plated (100 *μ*l) and CFU counted by eye after growth. For each biological replicate of ten capsules, values for three plating replicas were obtained and plotted in Fig. 4b.

To record luminescence and check for *V. fisheri* viability, Tecan Infinite M200 plate reader (luminescence mode) and NUNC 96 multiwell polystyrene white plates were used.

#### Capsule dry weight

To determine the dry weight of the alginate capsules, 5 replicates of 10 capsules, were created, weighed and left drying for overnight in Petri dishes open to air. 10 capsules were used for each assay to average over the capsule to capsule variation in weight and volume. The day after, each replicate was weighed a second time. The mean ratio of capsule weight reduction before and after drying was 0.05 w/w. For each organism 5, 10 capsules replicates, were analyzed.

#### Confirmation of sterile and aseptic conditions

To test for cross-contamination of the transport platform, 10 experiments of double transport (a capsule with and a capsule without cells on two different droplets) were performed. After the transport capsules were picked up using a sterile tweezer and placed in 5 ml of fresh medium. The capsules dissolved spontaneously in falcon tubes over few hours and the tubes were monitored for cell growth (exploiting O.D.). In addition the recovered samples were plated on selective agar plates and monitored for growth (CFU formation). As an additional test for sterility, aliquots from overnight cultures of all four organisms were directly incubated in 1-decanol or decanoate (10 *μ*l in 5 ml of decanoate or 1 ml of 1-decanol) for a time period corresponding to a typical transport step (5 minutes). To enable phase separation 4 ml of *Milli-Q* water were added in each falcon tube. Falcon tubes were centrifuged, supernatant discarded, and pellets resuspended in appropriate fresh media. Samples from the media were then plated and visually monitored for growth. *B. subtilis* and *E. coli* were plated on LB agar plates, *S. cerevisiae* on YPD plates and *V. fischeri* on LBS agar plates.

### Data availability

The datasets generated during and/or analysed during the current study are available from the corresponding author on reasonable request.

## Electronic supplementary material

Supplementary information
