# Supplementary material for: Transport of Live Cells Under Sterile Conditions Using a Chemotactic Droplet
Source: Sci Rep. 2018 May 30;8:8408. doi: 10.1038/s41598-018-26703-y (PMC5976712; doi:10.1038/s41598-018-26703-y)
Supplement: Supplementary file 1 — Supplementary information [file 41598_2018_26703_MOESM1_ESM.pdf]

Transport of Live Cells Under Sterile Conditions Using a Chemotactic Droplet

Silvia Holler[1], Carlotta Porcelli[1], Ioannis A. Ieropoulos[3], and Martin M. Hanczyc[1,2,*]

[1]Laboratory for Artificial Biology, Centre for Integrative Biology (CIBIO), University of Trento, 38123, Trento, Italy
[2]Chemical and Biological Engineering, University of New Mexico, MSC01 1120, Albuquerque, NM 87131-0001, USA
[3]Bristol BioEnergy Centre, Bristol Robotic Laboratory, Block T, UWE, Bristol, Coldharbour Lane, Bristol BS16 1QY, UK
[*]martin.hanczyc@unitn.it

Supplementary information

Supplementary Video S1

Supplementary Movie 1: Alginate capsule transport with live cells, 25x real time: https://www.youtube.com/watch?v=zCB2bPhFoCI. The chemotaxis experiment is performed in a glass Petri dish with diameter 9 cm with aqueous phase added, and the experiment is captured on video from above. Two 1-decanol droplets are placed in the experiment and each droplet has an alginate capsule of 1 mm diameter. 0 seconds (sec, s): droplets with capsules placed in experiment; 8 sec: salt gradient added with pipette (visible); 77 sec: end of droplet migration towards salt source; 85 sec: 0.2 M decanoate addition by pipette (visible) and capsule deposition; 148 sec: second salt addition; 227 sec: droplet migration towards second salt source.

Supplementary Figure S1

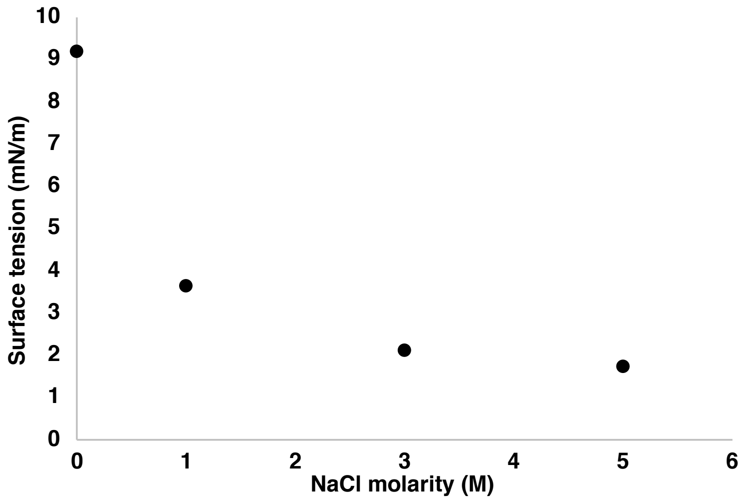

Surface tension of 1-decanol in different aqueous phases. Surface tension (in mN/m) for 1-decanol inverted pendant drop in: decanoate 5 mM pH 11.5 and decanoate 5 mM pH 11.5 mixed 1:1 with NaCl 1, 3, or 5 M. Error bars correspond to standard deviation of 10 replicates. Error bars not visible; standard deviation for each point ranged from 0.04 to 0.08 mN/m.

Supplementary Figure S2

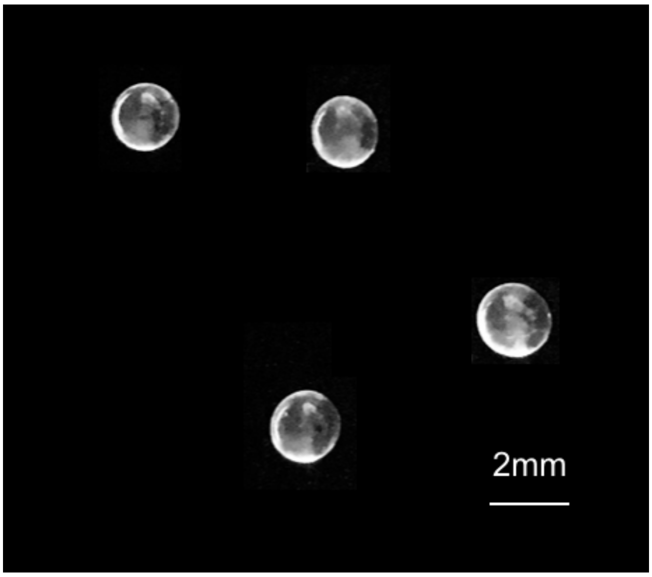

Alginate capsules Optical image, on black background, of alginate capsules (alginate dissolved in water 5 % w/v) just after crosslinking.

Supplementary Figure S3

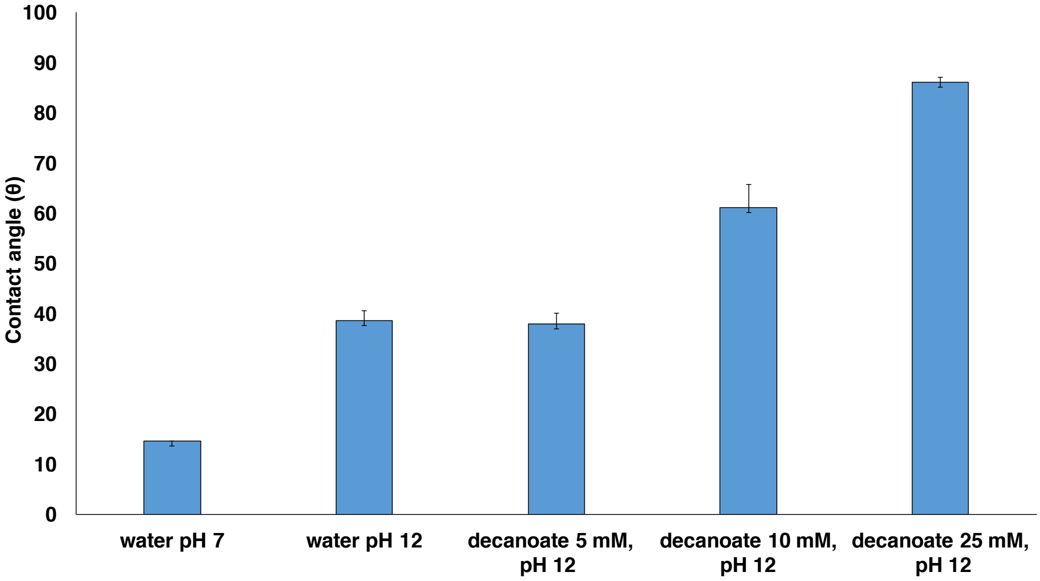

Contact angles of water on alginate hydrogels. Alginate hydrogel was prepared in various aqueous phases (water pH 7, water pH 12, decanoate 5 mM pH 12, decanoate 10 mM pH 12 and decanoate 25 mM pH 12) and 4 μl of water pH 7 deposited on top. Error bars correspond to standard error on five replicates.

Supplementary Figure S4

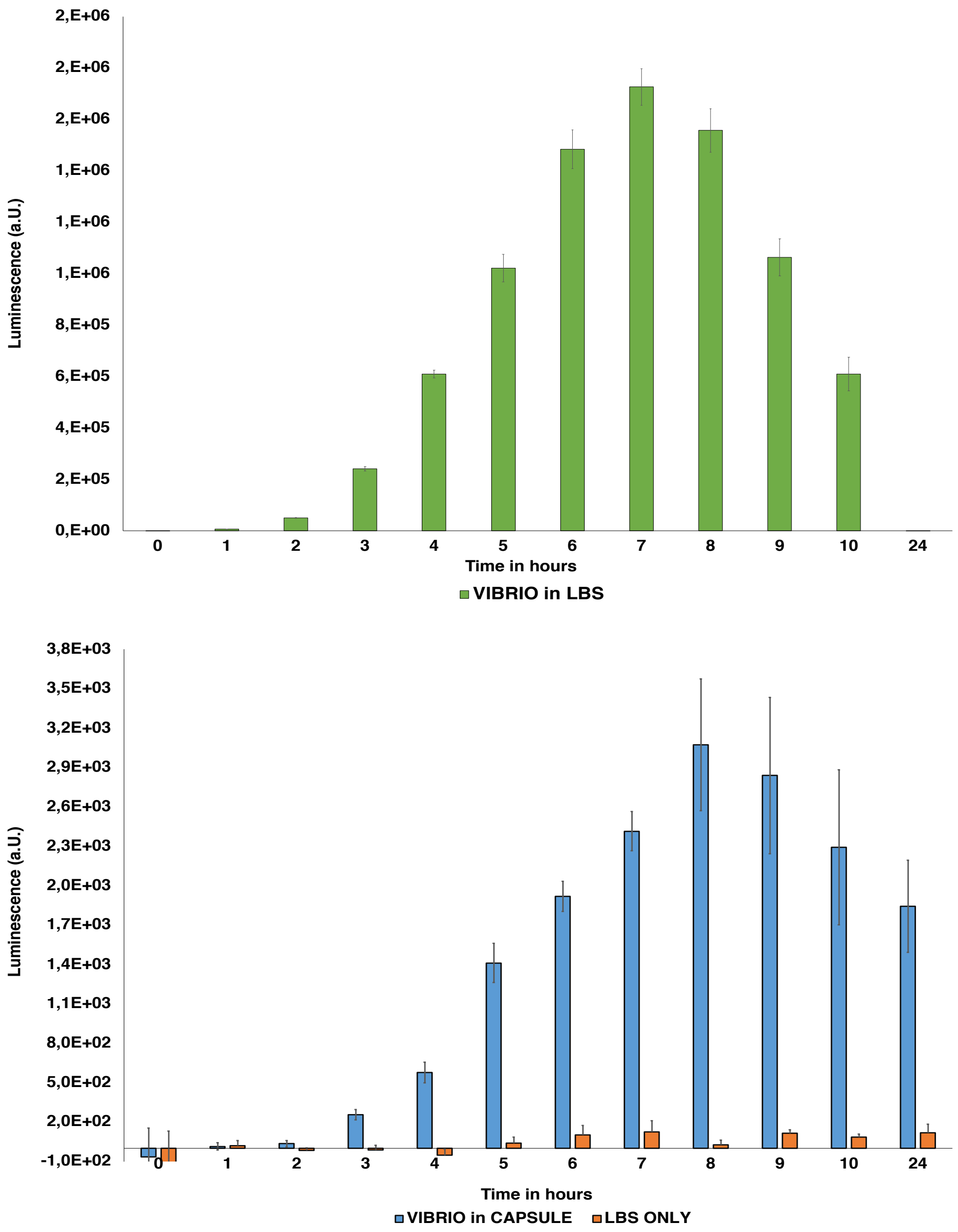

Autoluminescence of *V. fisheri* in alginate capsules. Luminescence values for *V. fisheri* with or without the alginate capsules over time. Top: luminescence profile over time for *V. fisheri* in LBS without capsule. 100 μl of sample was monitored with a starting O.D. at time 0 of 0.5. Bottom: luminescence profile over time for *V. fisheri* while placed in capsules and LBS alone as a background control. For *V. fisheri* in capsules: 10 μl, 0.2 X of the *V. fischeri* solution at time 0 mixed with alginate dissolved in water 5 % w/v. Time 0 is the time of *V. fischeri* encapsulation. Error bars correspond to standard errors of three technical replicates on three biological replicates of the same kind of sample (VIBRIO in LBS, VIBRIO in CAPSULES and LBS only). All the data were reported in arbitrary units (a.u.) and normalized with the subtraction of luminescence values obtained through the average value of three empty wells.
